# Lipid-Based Nanovectors for Targeting of CD44-Overexpressing Tumor Cells

**DOI:** 10.1155/2013/860780

**Published:** 2013-03-07

**Authors:** Silvia Arpicco, Giuseppe De Rosa, Elias Fattal

**Affiliations:** ^1^Dipartimento di Scienza e Tecnologia del Farmaco, University of Torino, Via Giuria 9, 10125 Torino, Italy; ^2^Dipartimento di Farmacia, University Federico II of Naples, Via Domenico Montesano 49, 80131 Napoli, Italy; ^3^Institut Galien Paris Sud, UMR CNRS 8612, University of Paris-Sud, 5 Rue Jean-Baptiste Clément, 92290 Châtenay-Malabry, France

## Abstract

Hyaluronic acid (HA) is a naturally occurring glycosaminoglycan that exists in living systems, and it is a major component of the extracellular matrix. The hyaluronic acid receptor CD44 is found at low levels on the surface of epithelial, haematopoietic, and neuronal cells and is overexpressed in many cancer cells particularly in tumour initiating cells. HA has been therefore used as ligand attached to HA-lipid-based nanovectors for the active targeting of small or large active molecules for the treatment of cancer. This paper describes the different approaches employed for the preparation, characterization, and evaluation of these potent delivery systems.

## 1. CD44 Receptor 

CD44 (*cluster of differentiation 44*) is a widely expressed cell surface hyaluronan receptor which consists in a single chain transmembrane glycoprotein with a size that varies between 80 and 200 kDa. It is moreover an acidic molecule with an isoelectric point between 4.2 and 5.8 [1]. CD44 receptor belongs to the family of cell adhesion molecules (CAMs) together with selectins, integrins, and cadherins. The CAMs control cell behavior by mediating contact between cells or between cells and the extracellular matrix and are essential for maintaining tissue integrity. Because of these important functions, they are also involved in pathological conditions including tumor progression and metastasis [2]. It is well known that various tumors, for example, epithelial, ovarian, colon, stomach, and acute leukemia, overexpress CD44 [3].

CD44 comprise a family of glycoproteins encoded by a single gene located on the short arm of chromosome 11 and composed of 20 exons [4]. Extensive alternative splicing generates multiple variant isoforms of CD44 receptor denoted as CD44v. The most abundant standard isoform of human CD44 protein is the smallest isoform that lacks any variant exons, designated CD44s, but some epithelial cells also express a larger isoform called CD44E [5]. The expression of CD44 isoforms containing combinations of the other variant exons is far more restricted in normal tissues. In particular, CD44s is abundantly expressed by both normal and cancer cells, whereas the variant CD44 isoforms (CD44v), that contain a variable number of exon insertions (v1–v10) at the proximal plasma membrane external region, are expressed mostly by cancer cells.

CD44 is endogenously expressed at low levels on various cell types of normal tissues [6, 7] but requires activation before binding to hyaluronan [8–11].

The CD44 structure of normal cells is distinct from that of cancer cells because pathological conditions promote alternate splicing and posttranslational modifications to produce diversified CD44 molecules with increased tumorigenicity [22, 23].

The effect of native hyaluronan as well as of the catabolic enzymes and the degradation products of this macromolecule on tumor progression is complex. Moreover, the amount of intratumoral hyaluronan also varies depending on the cell type and on the degree of tumor cell differentiation. There are some good reviews that describe the association of CD44 receptor with human cancer cells and underline the receptor's role in the progression of the disease [10, 24]; thus the overexpression of CD44 could be a good tool in drug delivery approaches using the receptor as an anchor to attach, through a ligand, prodrugs or nanomedicine-based delivery systems to increase the efficiency of anticancer drugs [25].

## 2. Hyaluronic Acid

**Figure 1:**
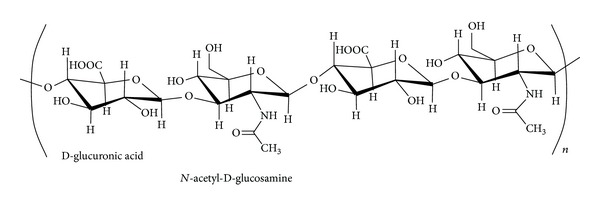
Chemical structure of HA.

Hyaluronic acid (hyaluronan, HA) is a nonsulfated glycosaminoglycan polymer. It is ubiquitous, being the main component of extracellular matrix [26]. HA is composed of disaccharide units of D-glucuronic acid and N-acetyl-D-glucosamine linked together through alternating *β*_1,3_ and *β*_1,4_ glycosidic bonds (Figure 1). HA is a biodegradable polymer with a molecular weight of 10^6^–10^7^ Da that is biocompatible, nontoxic, hydrophilic, and nonimmunogenic [27]. Moreover, HA molecules have a number of sites suitable for chemical modification such as hydroxyl, carboxyl, and *N*-acetyl groups.

In adult tissues such as the vitreous, synovial fluid and dermis, hyaluronan plays an extracellular, structural role that depends on its hydrodynamic properties as well as on its interactions with other extracellular matrix components. However, it is also concentrated in regions of high cell division and invasion (during embryonic morphogenesis, inflammation, wound repair, and cancer). Hyaluronic acid is thus also involved in tumorigenesis, and its role is complex and depends on various factors such as, for example its molecular weight. In fact lower molecular weight HA (10–100 kDa) stimulates angiogenesis but high molecular weight hyaluronan (>1,000 kDa) is inhibitory [28–30]. High amount of HA production usually promotes tumor progression, but it was observed that extremely high levels of hyaluronan production can be inhibitory [31]. As also reported, tumor progression is often correlated with both hyaluronan and hyaluronidase levels in human cancers [32]. These considerations led to the hypothesis that the turnover of HA is strictly involved in the promotion of tumor progression by HA [33–35].

In addition to its principal and previously described receptor, CD44, HA also interacts with other cell surface receptors such as RHAMM (receptor for hyaluronan-mediated motility, CD168), ICAM-1 (intracellular adhesion molecule-1), TLR-4 (toll-like receptor-4), HARE (HA receptor for endocytosis), and LYVE-1 (lymphatic vessel endocytic receptor).

The mechanism of HA-CD44 binding is still not fully understood, but it has been reported that the CD44 receptor contains the specific binding domain for HA, which consists of 160 amino acid residues. The binding affinity of CD44 to HA was found to be dependent on the size of HA oligomers. In fact, hexamer and decamer are considered to be the minimum size able to bind to CD44 while larger oligomers (20) have higher binding affinity because of their multiple interactions with more than one CD44 receptor simultaneously [3, 8, 36, 37]. 

It has also been reported that all the CD44 isoforms have uniform affinity for HA [38]; therefore HA can be used as vector for the active targeting of anticancer drugs. Different strategies have been exploited with interesting results, for example, in the preparation of bioconjugates obtained by covalently linking HA to a cytotoxic drug such as, for example paclitaxel [39, 40] or doxorubicin [41, 42]. These topics are out of the scope of this paper where only strategies consisting in the design of HA decorated nanosystems will be discussed in depth. 

## 3. Chemical Conjugation of HA to Lipid-Based Nanocarriers

Different approaches can be used to bind HA to the lipid-based nanocarriers, depending on the molecular weight of the HA as well as on the need to start from preformed nanocarriers or from pure lipids that will be then used to prepare particles.

HA binding to preformed nanocarriers was the firstly used method [43] and offers the advantage to conjugate the HA only on the external surface of the particle. Of course, this approach makes difficult the control of the density of attachment of HA on the carrier surface. Moreover, the lower specificity of the linkage, due to the possibility to bind different amino groups, results in a consequent multipoint attachment of the polymer on the nanocarrier that is then difficult to characterize.

Alternatively, HA can be previously conjugated to a pure lipid and then added in the lipid mixture during the preparation of the nanoparticles. This procedure permits the introduction of a controlled amount of HA on nanocarriers, but could require a more elaborated synthetic method.

### 3.1. HA Binding to Preformed Nanocarrier

**Figure 2:**
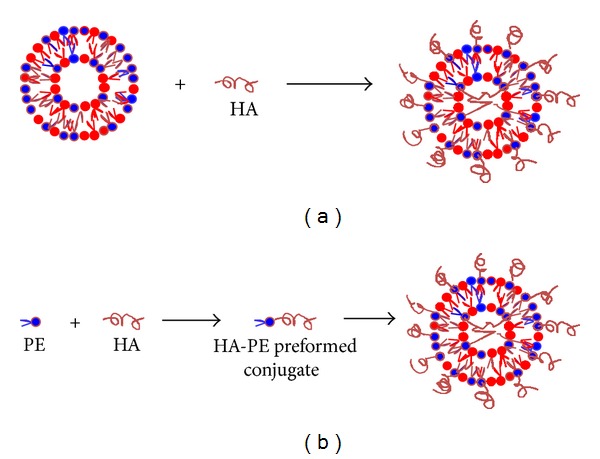
Strategies to prepare HA-coated nanocarriers. A schematic representation. (a) HA binding to preformed nanocarrier. Amidation reaction between HA-carboxyl group and aminoreactive group of lipid on the liposome surface. (b) Synthesis of HA-PE conjugates and following preparation of HA-coated lipid nanocarrier for postinsertion. (i) Reductive amination at the HA reducing end. (ii) Amidation reaction between HA-carboxyl group and aminoreactive group of lipid (PE).

High molecular weight (HMW) HA was attached to the surface of preformed liposomes through amidation reaction between the aminoreactive group of a lipid on the liposome surface, generally a phosphatidylethanolamine (PE), and HA glucuronic carboxylate (Figure 2) [13, 14, 43]. The amidation reaction was performed preactivating HA by incubation with the 1-ethyl-3-(3-dimethylaminopropyl) carbodiimide (EDC) condensing agent in acidic medium and then adding the activated HA to the nanocarrier suspension in a basic medium. Elimination of the excess of reagent and reaction byproducts was obtained by centrifugation and repeated washing.

### 3.2. Preparation of HA-PE Preformed Conjugates

HA conjugation to the lipid before nanocarrier preparation was carried out with both high and low molecular weight (LMW) polymers [12, 19]. In all cases, HA reacted with an aminoreactive group present on the lipid that was PE, also in this case (Figure 2). Two different conjugation methods have been proposed depending on the HA molecular weight. Eliaz and Szoka attached a mixture of oligosaccharide HA to PE by reductive amination using sodium cyanoborohydride as reducing agent [12]. Reductive amination is a chemical reaction widely used in polysaccharide conjugation and consists in two steps. In the first step, the aldehydic group of the terminal residue of HA, generated by opening the sugar ring, reacts, in acidic medium, with the amino group of PE forming the unstable imine. Then, the imine is reduced in the presence of a reducing agent to a secondary amine leading to the formation of the conjugate. An improvement of this reaction was proposed by the same group in 2006 [44]. The authors developed a methodology for the preparation of aldehyde functionalized HA and reported that the reductive amidination with this derivative is more efficient than that performed using the classical approach consisting in the reaction at the sugar reducing end.

In these reactions involving LMW-HA, only one PE molecule was linked to the polymer. Both kinds of conjugates were purified by silica column chromatography, and the latter was characterized by MALDI and ^1^HNMR.

HMW-HA-dioleoylphosphatidylethanolamine (DOPE) conjugate was prepared by EDC-mediated amidation reaction [19]. In this conjugate the DOPE amino group is randomly linked to the carboxylic residues of HA. The conjugate was purified by ultrafiltration and dialysis and its purity was assessed by capillary electrophoresis [20]. This conjugate was introduced into cationic lipids during liposome formation [19–21]. 

A similar synthetic approach was used by Toriyabe et al. [45] for the preparation of a conjugate between HA and stearylamine (HA-SA conjugate). SA was linked via an amide linkage using EDC and NHS as coupling agents; then the solution of conjugate was added and incubated to the liposome suspension.

Recently Cho et al. described the preparation of an amphiphilic polymer obtained conjugating HA oligomers to a cellular component, ceramide (CE). To obtain HA-CE conjugate, HA was first activated by reaction with tetra-n-butylammoniumhydroxide (HA-TBA), and CE was previously modified by esterification reaction with chloromethylbenzoyl chloride, used as linker. Then linker CE was conjugated to HA-TBA by ether bond formation [17].

## 4. Lipid-Based Nanocarriers for Targeting of CD44-Rich Cells

First evidence of powerful delivery of chemotherapeutics to cancer cells by HA-modified liposomes was provided by the group of Eliaz and Szoka [12] (Table 1). In this study, a low LMW-HA was bound onto the liposome surface. The authors demonstrated B16F10 cells expressing high levels of CD44, an avid cell-liposome binding followed by internalization in a temperature-dependent manner. Lower uptake was found in cells expressing low levels of CD44 (CV-1). B16F10 cell association of the unilamellar vesicles was found to depend critically on the density of HA on liposome surface. These findings were observed after exposing cells to HA-modified liposomes in both transient (3 h and replacement with fresh cell medium) and continuous conditions for periods going up to 24 h [12]. Moreover, for given amounts of intracellular-delivered chemotherapeutic agent, namely, doxorubicin (DOX), the encapsulated form was more efficient in killing B16F10 cells than the free form [12]. Due to the enhanced potency of DOX encapsulated into HA-modified liposomes, it was hypothesized that the drug reaches a critical compartment more efficiently, when compared with the free form. In particular, the authors hypothesized that an uptake of the delivery system via a non-clathrin-coated endosome, as already reported in the case of hyaluronan catabolism, could occur [46]. This hypothesis was recently confirmed by our group after incubating HA-modified cationic liposomes with CD44-expressing A549 cells with different endocytosis inhibitors [20]. It was found that the transfection efficiency of HA-modified cationic liposomes was not affected by a clathrin-mediated endocytosis inhibitor, while it was significantly decreased by inhibitors of caveolae-mediated endocytosis, demonstrating that the latter is the main endocytosis pathway of HA-bearing lipoplexes. It is worthy of note that in the studies of Eliaz et al. [47] and Dufaÿ Wojcicki et al. [20] an LMW and an HMW-HA were used, respectively, although a similar endocytotic pathway can be reasonably hypothesized.

The targeting of cancer cells using HMW-HA bound to liposomes was firstly demonstrated by Peer and Margalit [13, 14]. HMW-HA should offer advantages such as to bind the CD44 receptors with a higher affinity than hyaluronan fragments, to provide long-term circulation through its many hydroxyl residues, and to allow liposome lyophilization, due to the properties of HA to act as a cryoprotectant [48]. In particular, in an *in vivo* study, HA-modified liposomes resulted in long-circulating species, over a time frame at least equal to those reported for PEG-coated liposomes [13]. Mitomycin C (MMC), a chemotherapeutic agent used in different form of tumors but also characterized by severe side effects, was encapsulated into HA-modified liposomes and tested *in vitro* and in two experimental models of lung metastases. The *in vitro* studies showed that loading into the HA-modified liposomes generates a 100-fold increase in MMC potency in tumor cells that overexpress hyaluronan receptors, but not in cells with poor expression of these receptors. Moreover, when using HA-modified liposomes, MMC accumulated in the tumor 30-fold higher than when the drug was administered in free form and 4-fold higher than when delivered via unmodified liposomes. Interestingly, liver uptake was significantly reduced when the drug was delivered via the HA-modified liposomes that should contribute to reducing the subacute toxicity associated with MMC administered as free drug [13]. It is worthy of note that, in the case of MMC free or encapsulated in unmodified liposomes, tumor size, metastatic burden, and survival time were not much different than those observed in untreated mice. High positive responses were only reported in the case of mice treated with MMC HA-modified liposomes. Similar results were obtained from different experimental model of tumors with HA-modified liposomes, but replacing the MMC with DOX, thus demonstrating that the targeting is carrier-specific, rather than drug-specific [14]. In this study, the HA-modified formulation was compared to free DOX, DOX encapsulated in unmodified liposomes, and pegylated liposomes (Doxil). Drug accumulation in tumor-bearing lungs, as well as key indicators of therapeutic responses such as tumor progression, metastatic burden, and survival, was superior in animals receiving DOX-loaded HA-modified liposomes, compared to the controls. 

HA-modified lipid-based nanoparticles encapsulating paclitaxel (PXT) were also proposed. PXT is a chemotherapeutic agent largely used in the treatment of solid tumors. However, its poor water solubility as well as the lack of selective delivery approach represents important clinical limitations.* In vivo* evidence of CD44 targeting by HA-modified lipid-based nanoparticles was also obtained by encapsulating paclitaxel (PXT) into self-assembled lipid nanoparticle-like “clusters” [15]. Thus, HA-coated PXT-encapsulating clusters were administered in an experimental mice model of colon adenocarcinoma, and their antitumor effect, as well as the toxicity, was compared with that of FDA approved PXT formulations, namely, Taxol (PTX solubilized in the detergent Cremophor EL and in ethanol) and Abraxane (PXT encapsulated into albumin nanoparticles). Safety of the new HA-targeted formulation was demonstrated by any change in blood levels of enzymes released from the liver, namely, alanine aminotransferase (ALT) and aspartate aminotransferase (AST), respectively, regarded as reliable indicators of liver tissue damage and, more generally, systemic tissue damage. This effect was not associated with any change in body weight. On the contrary, multiple i.v. administrations of Taxol resulted in changes of body weight and release of high amounts of liver enzymes [15]. Moreover, when using Taxol, PXT was eliminated from the circulation within less than 1 h after i.v. injection, while PTX, administered within HA-modified lipid clusters, was still circulating even 24 h after i.v. injection. These findings still support the hypothesis that HMW-HA, when used as targeting moieties, also confers stealth properties on the nanoparticles. Interestingly, the HA-modified nanoparticles reduced PTX liver and spleen accumulation by almost 2-fold and increased PTX accumulation in the tumor by 10-fold compared to Taxol. Finally, tumor progression was exponential in the case of 5 mg/Kg body Taxol or Abraxane, while it was arrested at the same dose of PXT administered in HA-modified lipid clusters. This effect was also obtained with 20 mg/Kg body of Taxol, although it was associated with a significant loss of body weight indicating global toxicity [15]. Recently, Yang et al. proposed the preparation of HA-coated nanostructured lipid carriers (HA-NLCs) for tumor targeting via electrostatic attraction [16]. In this approach, cationic NLCs loaded with PTX were prepared by melt emulsion technology, followed by coating with HA (300 kDa); the process of electrostatic attraction was simple and controllable, and no chemical reagents were needed. The *in vitro* cytotoxicity and *in vivo* antitumoral activity studies showed that HA-PTX-NLCs were more effective than Taxol with fewer side effects. HA-NCL also prolonged the blood circulation time of PTX and increased its accumulation in tumors.

HA-modified nanoparticles have been proposed to overcome clinical limits of chemotherapeutics, such as Docetaxel (DCT). DTC is a semisynthetic taxane derivative very effective against different tumors, but its clinical use causes several side effects and other limitations regarding the appearance of multidrug resistance (MDR) and its insolubility. Recently Cho et al. described the preparation of HA-ceramide (CE) self-assembled nanoparticles for DCT and DOX active targeting [17, 49]. The *in vitro* cellular uptake studies showed that nanoparticles enhanced intracellular DCT uptake in the CD44-overexpressing cell lines MCF-7. MDR-overcoming effects of DCT nanoparticles were observed in cytotoxicity test in CD44-positive MCF-7 breast cancer cells resistant to doxorubicin. The *in vivo* tumor targetability was evaluated using a noninvasive fluorescence imaging system in the same cells xenografted in a mouse model. To assess the uptake mechanism by a competitive inhibition assay, CD44 receptors were blocked with preinjection of high doses of HA. The fluorescence signal in the HA preinjected animal group was lower than that in no preinjection group for 24 h, indicating a probable reduction in nanoparticle uptake due to the blocking of CD44. The real-time imaging data showed that the fluorescent signal increased for the first 6 h and was maintained for 1 day. Then the tumors were dissected 24 h following injection, and the observed fluorescence intensity of HA pre-injection group was only 43.9% of the no preinjection group.

The same research team described the preparation of DOX-loaded, self-assembled, HA-CE-PEG-based nanoparticles [18]. *In vitro *tests were performed on two different cell lines with different CD44 expression: NIH3T3 (mouse embryonic fibroblast cells, CD44-negative) and SCC7 (mouse squamous cell carcinoma cells, CD44-positive). The cytotoxicity studies showed that HA-CE-based nanoparticles can be used as vehicle without important toxicity. The cellular uptake efficacy of DOX from nanoparticles via HA and CD44 interaction was demonstrated by confocal microscopy analysis. *In vivo *studies on SCC7 tumor xenograft mouse model showed improved retention time in the bloodstream and nanoparticle accumulation at the tumor site. The pharmacokinetics evaluation confirmed that PEGylation resulted in prolonged nanoparticle circulation and reduced DOX clearance rate. Improved half-life of DOX, when formulated as HA-CE-PEG nanoparticles, led to higher *in vivo* antitumor efficacy in the tumor xenograft mouse model in comparison to non-PEGylated nanoparticles and DOX alone.

HA was also used to increase transfection efficiency of cationic liposomes. Plasmid DNA and siRNA were successfully delivered to CD44-expressing cancer cells with this approach [19, 21]. The use of a lipid conjugate HA-DOPE into the liposome composition did not affect the lipoplex formation upon liposome mixing with DNA [19] or siRNA [21]. On the contrary the lipoplex zeta potential was strongly affected shifting from a positive to a negative value. This was consistent with the presence of HA at lipoplex surface. Moreover, the presence of HA in the liposome formulation led to increased nucleic acid protection from degradation against DNase I or RNAse V1, probably because the HMW-HA and cationic lipids prevent access of these enzymes to the whole colloidal system [19, 21]. The presence of HA-DOPE did not modify the *in vitro* cytotoxicity, on the MDA-MB-231 and MCF-7 breast cancer cell lines, characterized by high and low expressions of CD44, respectively. On the contrary, the use of HA strongly reduced the cytotoxic profile of DOTAP/DOPE liposomes in combination with siRNA on A549 CD44-expressing cells [21]. This effect was attributed to the endogenous nature of HA that should be biocompatible and, when located on the lipoplex surface, might avoid the direct contact of the cationic liposome with the negatively charged cell surface and hence reduce its cytotoxic potential. Finally, HA-DOPE increased the level of transfection on CD44-highly expressing cells (MDA-MB-231 or A549) compared to the cells expressing low levels of CD44 (MCF-7 or Calu-3). The involvement of the CD44 receptors was confirmed by using anti-CD44 Hermes-1 antibody that highly inhibited transfection efficiency; this effect was not observed by nonspecific anti-ErbB2 antibody [19, 20].

HA-coated cationic liposomes were also prepared using an HA-stearylamine (SA) conjugate, and their ability to reach liver endothelial cells was evaluated [45]. The pharmacokinetics and biodistribution studies on HA-SA modified liposomes showed that liver accumulation was higher than the corresponding value for nonmodified liposomes at every time point and increased depending on the extent of modification of HA-SA. On the contrary, if free HA was introduced on liposomes surface, via electrostatic interactions, liver accumulation decreased indicating that HA alone did not fully function as targeting ligand. From confocal microscopy analysis, HA-SA modified liposomes accumulated along the blood vessels to a greater extent than nonmodified liposomes, suggesting that the HA-coated liposomes are distributed within endothelial cells in the liver.

Recently, the complement activation capacity of HA nanoparticles has been investigated [20, 50]. Complement activation is an important aspect to consider since it may initiate adverse reactions among sensitive individuals and could represent an obstacle for the clinical application of HA-decorated nanovectors. Mizrahy et al. evaluated the level of the terminal complement pathway activation markers C5a and SC5b-9 by ELISA on a panel of nanoparticles decorated with HA of different molecular weights (ranging from 6.4 kDa to 1500 kDa). In these experiments, no induction of complement activation was observed, and the marker levels remained comparable with the baseline value [50]. Dufaÿ Wojcicki et al. [20] evaluated the behavior of HA lipoplexes made with increasing lipids : DNA ratio (2, 4, and 6) and the activation of a protein of complement cascade, the protein C3, were determined by 2D immunoelectrophoresis. Low activation of complement was observed in all the formulations although lipoplexes containing HA with lipids, DNA ratios of 4 and 6, give higher values than the respective nonhyaluronate samples [20]. These data suggest that HA-coated nanosystems could be an interesting alternative to PEG grafted particles since the latter were shown to activate complement after intravenous administration [51].

The impact of HA size and density of HA-grafted nanoparticles on affinity toward CD44 was evaluated in a systematic manner [50, 52]. Qhattal and Liu prepared liposomes decorated with HA of both low and high molecular weights (5–8, 10–12, 175–350, and 1600 kDa) and with different degree of grafting density. They performed *in vitro* studies (fluorescence microscopy analysis, flow cytometric analysis, and competitive binding experiments) and stated that cellular targeting efficiency of HA liposomes depends on HA molecular weight, grafting density, and cell surface CD44 receptor density. In particular, the HA liposomes binding and internalization increased with increasing polymer molecular weight and/or the grafting density [52]. A small library of HA-coated nanoparticles distinguished by the size of their surface HA was also described [50]. The authors used HA of 5 different molecular weights (6.4 kDa, 31 kDa, 132 kDa, 700 kDa, and 1500 kDa) and evaluated the nanoparticles interaction with CD44 receptor through surface plasmon resonance analysis. Also in this case, the affinity towards CD44 was low for LMW-HA and increased with the polymer molecular weight [50].

## 5. Conclusions

HA represents a promising opportunity to develop new cancer therapies. A growing number of scientific works explored the possibility to target cancer cells overexpressing CD44 receptor by using HA-modified vectors. HA is biocompatible, biodegradable, nontoxic and noninflammatory. Moreover, it can easily undergo chemical modifications and conjugates with drugs or other ligands. HA targeting of cancer cells overexpressing CD44 receptor has been largely demonstrated. In addition, HA coating has been recently proposed as a safer alternative to PEG grafting in order to increase the particles' half-life. The success of this strategy is demonstrated by an HA conjugate at the moment in clinical trials. A phase III clinical trial based on a hyaluronic acid-Irinotecan conjugate is in the recruitment state, and the final data collection is scheduled for January 2014. The possibility to conjugate HA to lipid-based nanocarriers, such liposomes that are on long time in the clinical practice, should open new opportunities to target cancer cells also with drug that cannot be easily conjugated to HA. Further studies are certainly needed to understand the relations between the molecular weight and “biological” properties of HA, especially in the interaction of HA-modified nanoparticles with the target.

Moreover, further information on the *in vivo* distribution of HA conjugated nanocarries as well as their tumor localization should be useful to design new anticancer therapies based on CD44 targeting. 

## Figures and Tables

**Table 1:** Examples of HA-decorated lipid-based nanocarriers for targeting of CD44.

Carrier	Drug	HA	Main findings	Reference
Liposomes	DOX	LMW-HA	Avid cell-liposome binding followed by internalization in cells overexpressing CD44. Higher cytotoxicity compared with free drug on CD44-overexpressing cells.	[12]
Liposomes	MMC DOX	HMW-HA	Higher affinity of HMW-HA to bind the CD44 receptors, compared to hyaluronan fragments. Long-term circulation of HMW-HA liposomes. HMW-HA can act as cryoprotectant, thus allowing liposome lyophilization. Loading into the HA-modified liposomes generates a 100-fold increase in drug potency in tumor cells overexpressing CD44 receptors. Higher drug accumulation in tumor, compared to free drug or drug in unmodified liposomes.	[13, 14]
Self-assembled lipid nanoparticles	PTX	HMW-HA	Reduced PTX accumulation in liver and spleen and increased drug accumulation in the tumor, compared to Taxol. Prolonged PTX half-life. Reduced PTX toxicity.	[15]
HA-coated nanostructured lipid carriers	PTX	HMW-HA	More effective than Taxol with fewer side effects. Prolonged PTX half-life. Increased PTX accumulation in tumors.	[16]
Self-assembled nanoparticles	DCT	LMW-HA	Enhanced intracellular DCT uptake in the CD44-overexpressing cell lines. MDR-overcoming effects. *In vivo* specific CD44-mediated tumor targeting.	[17]
PEGylated self-assembled nanoparticles	DOX		Improved retention time in the bloodstream and nanoparticle accumulation at the tumor site. PEGylation resulted in prolonged nanoparticle circulation and reduced DOX clearance rate. Higher *in vivo* antitumor efficacy in the tumor xenograft mouse model in comparison to non-PEGylated nanoparticles and DOX alone.	[18]
Cationic liposomes	DNA and siRNA	HMW-HA	The presence of HA-DOPE lipid conjugate in the liposome composition did not affect the lipoplex formation. Increased nucleic acid protection against enzymatic degradation. Increased the level of transfection on CD44-highly expressing cells.	[19–21]
Nanoparticles	—	Different molecular weights	No induction of complement activation.	[18]
